# Psychosocial Characteristics of Totally Blind People in a Nigerian City

**DOI:** 10.4103/0974-9233.71603

**Published:** 2010

**Authors:** Dupe S. Ademola-Popoola, Mosunmola F. Tunde-Ayinmode, Tanimola M. Akande

**Affiliations:** Department of Ophthalmology, University of Ilorin Teaching Hospital, Ilorin, Nigeria; 1 Department of Behavioural Sciences, University of Ilorin Teaching Hospital, Ilorin, Nigeria; 2 Department of Epidemiology and Community Health, University of Ilorin Teaching Hospital, Ilorin, Nigeria

**Keywords:** Blind, Population, Psychosocial Characteristics

## Abstract

**Purpose:**

To characterize the demographic and psychosocial problems of a group of blind people as a way of attracting more attention to and providing data that can improve the psychosocial care of the visually impaired.

**Materials and Method:**

A cross-sectional descriptive study of a population of totally blind people in Ilorin, Nigeria using a self-report questionnaire (SRQ). The questionnaire was verbally administered by the study personnel in the local language. Simple frequency tables were obtained and the Chi-square test was performed to determine significant differences between variables. *P* value <0.05 was considered statistically significant.

**Results:**

Sixty one blind patients consented to participate. Most participants were engaged in street begging for their livelihood. Most subjects desired a job change, signifying dissatisfaction with the present occupation. Up to 80% of the cohort was married and had spouses who were also blind in at least one eye. Approximately two-thirds had five or more children and majority lived with family members who were responsible for taking care of their personal hygiene, cooking and mobility. The majority developed blindness in childhood and 16% had a family history of blindness and 77% had never used conventional eye care, with corneal disease being the most frequent cause of blindness. Many feared that their children may also become blind. Thirty-one (51%) scored ≥5 on SRQ and were classified as probable cases of psychological disorder.

**Conclusion:**

Blindness in a majority of cases that started in childhood was probably preventable. Inaccessibility to or failure of the formal rehabilitation and social welfare systems may have caused this psychosocial dilemma. The high level of social and family interaction provides opportunity for organized preventive ophthalmology, community health care services and psychosocial care.

## INTRODUCTION

Blindness is a major health burden globally, yet more prevalent in developing countries such as Nigeria where childhood infections and malnutrition are rife and eye care services are inadequate.^1^ The World Health Organization (WHO) defines blindness as visual acuity <3/60 or corresponding visual field loss to <10° in the better eye with best correction.^2^,^3^ Visual impairment represents low vision and blindness. The WHO defines low vision as visual acuity of <6/18 but equal or better than 3/60 in the better eye with best correction. It is estimated that there are approximately 37 million blind people globally, with an overwhelming majority residing in the developing world. However, 75% of all blindness is avoidable.^3^

The current burden of blindness in Nigeria is estimated at 1 million blind adults and 3 million visually impaired people. Approximately two of every three blind people have an avoidable cause of blindness.^4^

The global average prevalence of blindness is estimated at 0.7%, ranging from 0.3% in the developed countries to 1.4% in the developing countries.^2^ However, in Nigeria, the prevalence may vary from region to region,^4^–^8^ and the national average is estimated at 1%.^5^

Strikingly, blindness in Nigeria is three-times more prevalent in the dry Northern Sahel region than in the Southern Delta region. Blindness in Nigeria has also been associated with the level of illiteracy, which is higher in Northern Nigeria than in Southern Nigeria.^4^

While cataract is the most common cause of blindness in Nigeria, accounting for more than one-third of the cases, glaucoma, corneal scarring and poor procedures for cataract surgery also contribute to the burden of blindness.^4^–^8^ The contribution of river blindness or onchocerciasis and trachoma is also substantial, accounting for roughly 5% of the blindness in Nigeria.^4^

About 82% of the blind globally are 50 years of age or older,^3^ which is similar for the blind in Nigeria.^4^–^6^,^9^

Globally, there has been a concerted effort at tackling the burden of blindness, which is well articulated in “Vision 2020: Right to Sight.”^10^ This global initiative to eliminate avoidable blindness by the year 2020 is a partnership between the WHO and the International Agency for the prevention of blindness.^10^

Consistent with this strategy, there has been considerable focus on the identification and treatment of blinding diseases in Nigeria. However, a large group of pre-existing, irreversibly blind individuals are yearning for improved quality of care and regular care in Nigeria. The rehabilitation of this group remains a major challenge for the Nigerian society.

The psychosocial care of the existing blind in Nigeria may not have received enough attention. Educational institutions to provide the blind with daily living skills are rare and generally not adequately equipped in terms of infrastructure and manpower.^11^,^12^ Hence, many blind people have been isolated or disconnected from their communities and families because of adjustment problems or the search for a better life. A number of the blind end up as street beggars in major cities or towns, especially those without education or vocational training.^4^,^13^

The frustration of the blind in Nigeria is palpable; they are disadvantaged in terms of education, vocational training, mobility and socioeconomic activities.^13^–^15^ Blind people generally find it problematic to attend school, gain employment and to be part of community activities. This may be a by-product of the fact that blindness still evokes negative attitudes and some degree of stigma in society.^13^,^16^ Undoubtedly, lack of education and inadequate rehabilitation services make many of the blind eventually resort to street begging, resulting in a poor quality of life.

In Nigeria, begging and blindness are closely associated, with 70% of the 4,000 beggars in Lagos being blind.^17^ Although begging has become a social menace in Nigeria, it is thought to have religious, cultural and social roots, particularly in the Northern part of the country, where it is more prevalent. In this cultural setting, beggars form well-organized communities and even have Chiefs or Heads.^18^ Begging also carries along with it a social stigma, particularly in Southern Nigeria.^18^

Whereas the physical, psychological and socioeconomic distress of blindness is well recognized,^1^,^5^,^15^,^19^,^20^ in view of the relative paucity of literature, the attitude of the medical community in Nigeria toward psychosocial care of the blind can be described as unenthusiastic.

This study is an attempt to characterize the demographic and psychosocial problems of a group of blind people in Nigeria as a way of attracting more attention to and providing information that can improve the psychosocial care of the visually impaired, generally.

## MATERIALS AND METHOD

### Study design and population

This was a cross-sectional descriptive study of a population of totally blind people in Ilorin, Nigeria, over a period of 2 months. The study population was composed of two groups: a group of blind people living in a suburban area of the town (SBC group) and another belonging to a blind people association in the town (BA group).

### Location and background

The study was carried out in Ilorin, the capital city of Kwara state, one of the 36 states in Nigeria located in the North central zone of the country. Ilorin is inhabited predominantly by the Yorubas, one of the major ethnic groups in Nigeria. The Indigenes are predominantly Muslims. One of the groups resided in the suburban area of the city where the majority of indigenous people lived. This group lives communally in a separate area with a chief or head who oversees the day to day running of the community. The BA group lived in their homes within the town and had a regular meeting place, which made their investigation easy.

### Sampling and data collection

The blind people were selected consecutively in the communities where they lived or gathered for their monthly meetings based on their willingness and consent to participate in the study.

The sociodemographic data of the cohort and the characteristics in relation to blindness were collected using a semi-structured questionnaire designed by the authors. Additionally, the 20-item self-reporting questionnaire (SRQ-I) was used to assess the cohort for probable psychological disorder.^21^ All questionnaires used in the current study were oral questionnaires, which were administered verbally by the study personnel. All responses were recorded by the study personnel.

A proforma questionnaire was designed to gather information relating to their personal data, marriage and children, nature of blindness, views on education for blind people, family support, requirement for lead or assistant, handling of money and daily living activities.

The SRQ was constructed for use in the WHO study on strategies for extending mental health care.^21^ It is specifically designed for screening of psychiatric disturbances in the primary care setting.^21^ The non-psychotic section containing 20 items (SRQ-I) was used. Each item in the questionnaire has a score of 0 or 1 for No or Yes responses, respectively.^21^ SRQ-I was validated in a primary care setting in rural Southwest Nigeria and found to effectively discriminate between patients with and without psychiatric morbidity.^22^ This was best done at a cut-off point of 5, which has the optimal sensitivity of 98.8% and specificity of 90.9%.^22^ This cut-off was adapted for this study because of the similarities in clinical setting, culture and language.

Pre-study visits were made to both groups to seek their participation in the study. For the SBC group, their chief was approached and we were given his assurance of cooperation in enlisting subjects. The BA group leader was approached in a similar manner. The BA group lived in the more urban or developed areas of the city. The cooperation of the SBC group was secured on the condition that data could only be collected in their homes and on Sundays, when they would be relaxing, and while a member of the association assisted in recruiting participants. Subjects in the BA group were interviewed at their meeting place after their regular meetings.

Only those who gave their consent to participate were interviewed by the investigating team consisting of two of the authors and two trained research assistants who spoke Hausa language fluently. The questionnaire was read aloud to each of participants in the appropriate language.

Ophthalmic examination was carried out by one of the authors (Ophthalmologist) assisted by the research assistants. Firstly, attempts were made to assess visual acuity with Snellen chart. Secondly, further examination was performed to detect the cause of the visual loss. Anterior segments were examined with a loupe and, where the media allowed for it, the fundus was examined using a direct ophthalmoscope. Visual loss was categorized according to the WHO definition of visual impairment.^2^,^3^

### Data analysis

Data analysis was conducted using EPI-info version 6 (Centres for Disease Control and Prevention, USA). Simple frequency tables were obtained and the chi-square test was performed to determine significant differences between variables. A *P*-value <0.05 was considered statistically significant.

## RESULTS

### Sociodemographic characteristics

There were 61 blind people who consented to be interviewed, 44 (72%) from the SBC group and 17 (28%) from the BA group. There were 54 (89%) males and seven (11%) females [Table 1]. The majority (56%) were aged 45 years and older, with a mean age of 40.72 ± 14.23 years [Table 1]. In the cohort, 57% had an arabic education only while 28% had a western education [Table 1]. Most were street beggars (72%) and only 10% were engaged in skilled jobs. Most of the cohort (80%) was married, most of their spouses were also blind (74%) in at least one eye and 64% had five or more children [Table 1].

**Table 1:** Sociodemographic characteristics of the study population

Variables	*N* = 61	
	*n*	%
Gender		
Male	54	89
Female	7	11
Age group in years		
15–30	21	34
31–45	8	13
≥45	32	56
Ethnic group		
Hausa/Fulani	43	70
Yoruba	14	23
Others	4	7
Religion		
Islam	54	89
Christianity	7	11
Educational status		
No form of education	9	15
Arabic education only	35	57
Some form of western education	17	28
Occupational status		
Street begging	47	77
Other unskilled jobs	5	8
Semiskilled job	2	3
Skilled jobs	7	12
Marital status		
In a marriage	45	80
Not in a marriage	16	20
Duration of marriage in years		
≤10	13	21
≥10	32	53
Not applicable	16	26
Mode of contracting marriage		
Self	31	51
Family	14	23
Not applicable	16	26
Type of family		
Monogamy	22	36
Polygamy	23	38
Not applicable	16	26
Blindness in spouse(s)		
Not blind	11	18
Blind in one eye	32	53
Blindness in both eyes	2	3
Not applicable	16	26
Number of children		
<5	29	48
≥5	16	26
Not applicable	16	26
Living arrangement		
Alone	15	25
With spouse(s)	24	39
With other relatives	22	36

*N* = number people in the study population who responded, n = number of people in the sample population affected by the variable, % = percentage of *n/N* to the nearest whole number, Not applicable = these variables did not apply to those who are “not in a marriage”

### Characteristics of blindness

The mean duration of blindness was 25.90 ± 16.37 years and the majority (72%) had been blind for 20 or more years; 62% developed blindness in childhood while 21% became blind in adult life. Also, 16% had a family history of blindness [Table 2]. Several of the subjects (77%) had never used orthodox eye treatment and many feared that their children may also become blind. Corneal disease was the most frequent cause of blindness.

**Table 2:** Characteristics of blindness in the study population

Variables	*N* = 61	
	*n*	%
Duration of blindness in years			
0–10	8	13	
10–20	9	15	
20–30	18	30	
≥31	26	42	
Age at which blindness occurred in years			
Since birth	10	16	
0–20	38	62	
21–40	7	12	
≥41	6	10	
Family history of blindness			
Yes	10	16	
No	51	84	
History of previous orthodox treatment for blindness	14	23	
Yes	47	77	
No			
Hope for a cure			
Yes	37	61	
No	24	39	
Fear of children getting blind			
Yes	28	46	
No	33	54	
Cause of blindness			
Corneal problem	47	83	
Lens problem	4	7	
Glaucoma	6	10	
Others*	4	7	

*N* = number people in the study population who responded, *n* = number of people in the sample population affected by the variable, % = percentage of *n/N* to the nearest whole number,

* Others = whole eyes involved, e.g. phthisis bulbi, trauma, evisceration

### Social characteristics

In economic terms, about half earned <250 naira/day, which is equivalent to approximately $1.50 in American currency [Table 3]. A large proportion of the cohort desired a change in their job [Table 3]. The majority managed their own finances despite indicating that they could trust people with their money, but support from extended family was only available to some of the cohort [Table 3]. A few people had other diseases co-morbid with blindness, which included high blood pressure, osteoarthritis, etc. Thirty-one subjects (51%) scored ≥5 on the SRQ and were classified as probable cases of psychological disorder.

**Table 3:** Social adjustment characteristics of the blind study population

Variables	N = 61
	*n*	%
Average income per day in Naira		
≤150	21	34
151–250	14	23
251–500	12	20
>500	14	23
Like a change of job		
Yes	54	89
No	7	11
Blind person should be educated		
Yes	52	85
No	9	15
Knows a school for the blind		
Yes	53	87
No	8	13
Had vocational training		
Yes	14	23
No	47	77
Receives support from extended family		
Yes	15	25
No	46	75
Recognition of people by		
Voice	42	69
Touch	7	12
Voice and touch	10	16
Residual vision	2	3
Requires a lead		
Yes	59	81
No	2	19
Person who lead		
Children	10	16
Spouse	34	56
Relatives	13	21
Commercial hands	4	7
How money is kept		
Self	52	85
Spouse	6	10
Children	2	3
Relatives	1	2
Trust others with money		
Yes	30	49
No	31	51
Who does cooking		
Spouse/children	25	41
Relatives	15	25
Food vendors	21	32
Personal hygiene cared by		
Spouse/children	39	64
Relatives	6	10
Commercial hands	16	26
SRQ score		
<5	30	49
≥5	31	51
Has other disease		
Yes	10	16
No	51	84

*N* = total number of people in the study population that responded, *n* = number of people in sample population affected by variable, % =percentage of *n/N* to the nearest whole number

### Comparison of the SBC group and the BA group

The BA group was younger, had fewer married subjects (21% versus 89%) and enjoyed more family support (86% versus 6%) [Table 4]. The BA group was also better educated, earned above 500 naira per day and had received orthodox eye treatment [Table 4]. The BA group also had higher number of people who received vocational training (50% versus 15%). The BA group were less likely to have a psychological disorder compared with the SBC group (29% versus 57%, respectively) [Table 4].

**Table 4:** Comparison of the BA group with the SBC group

Variables	Total *N* = 61	SBC group *N_1_* = 47 *n_1_* (%)	BA group *N_2_* = 14 *n_2_* (%)
Age distribution (years)			
15–30	21 (34)	13 (28)	8 (57)
31–45	8 (13)	4 (9)	4 (29)
≥45	32 (56)	30 (64)	2 (14)
Gender distribution			
Male	54 (89)	46 (98)	8 (57)
Female	7 (11)	1 (2)	6 (43)
Religion			
Islam	54 (89)	47 (100)	7 (50)
Christianity	7 (11)	0 (0)	7 (50)
Educational status			
No education	9 (15)	9 (19)	0 (0)
Arabic education only	35 (57)	35 (74)	0 (0)
Primary education	4 (7)	3 (6)	1 (7)
Secondary education	6 (10)	0 (0)	6 (43)
Tertiary education	7 (11)	0 (0)	7 (50)
Occupational status			
Street begging^*^	47 (77)	47 (100)	0 (0)
Other unskilled jobs	5 (8)	0 (0)	5 (38)
Semiskilled jobs	2 (3)	0 (0)	2 (14)
Skilled jobs	7 (12)	0 (0)	7 (50)
Ethnic group			
Hausa/Fulani	43 (70)	43 (91)	0 (0)
Yoruba	14 (23)	0 (0)	14 (100)
Others^*^	4 (7)	4 (9)	0 (0)
Marital status			
In a marriage	45 (80)	42 (89)	3 (21)
Not in a marriage	16 (20)	5 (11)	11 (79)
Previous orthodox eye treatment			
Yes	14 (23)	0 (0)	14 (100)
No	47 (77)	47 (100)	0 (0)
Feelings despite being blind			
Happy	17 (28)	10 (59)	7 (41)
Sad	33 (54)	28 (85)	5 (15)
Indifferent	11 (18)	9 (82)	2 (18)
Hopes for a cure			
Yes	37 (61)	28 (60)	9 (64)
No	24 (39)	19 (40)	5 (36)
Average income per day in Naira			
<500	47 (77)	47 (100)	0 (0)
>501	14 (23)	0 (0)	14 (100)
Would like to change job			
Yes	54 (89)	43 (91)	11 (79)
No	7 (11)	4 (9)	3 (21)
Has vocational training			
Yes	14 (23)	7 (15)	7 (50)
No	47 (77)	40 (85)	7 (50)
Receives support from extended family			
Yes	15 (25)	3 (6)	12 (86)
No	46 (75)	44 (94)	2 (14)
SRQ score			
<5	30 (49)	20 (43)	10 (71)
≥5	31 (51)	27 (57)	4 (29)
Has another disease			
Yes	10 (16)	9 (19)	1 (7)
No	51 (84)	38 (81)	13 (93)

*N* = the total number of people in the study population, *N*_1_ = the total number of the SBC, *N*_2_= the total number of the BA, Percentages in column brackets to the nearest whole number

## DISCUSSION

The psychosocial dimension of blindness is not often properly addressed by eye specialists, especially in developing countries.^11^ This may partially be due to the heavy work load of physicians, poor rehabilitation facilities and inadequate opportunities available for people with disability in Nigeria. Ophthalmologists must be aware of both the social and the emotional impact of blindness and support services available in the community.^19^,^23^ Nevertheless, psychosocial care will develop only if the circumstances of the blind are properly understood and investigated. The data from our study contribute to this endeavour.

Small blind community formation, especially in northern Nigeria, is not uncommon. This study, for example, included an organized suburban community of blind people (SBC) and a blind people association (BA). These types of organizations give the blind opportunity to support one another, build a psychosocial front against stress, develop coping skills and engage in quality relationships, which may occasionally lead to marriage. It is also possible to deliver preventive and curative health care, especially ophthalmic care, in this kind of a setting. Even if these people are blind, their children, spouses and relatives who are not can enjoy preventive eye care. These blind people need be educated to the fact that their condition could have been prevented and that they should prevent occurrence in their children. Efforts must be made to reinforce good nutrition, immunization and basic eye hygiene to prevent blindness.

The cause of blindness in this cohort was largely corneal disease rather than lenticular pathology. This is in contrast to global data, which indicate lenticular pathology as the most common cause of blindness followed by glaucoma.^2^ This outcome indicates that the age of onset of blindness was likely during childhood, and was most probably avoidable. Most childhood blindness is preventable through basic eye care, immunization, particularly measles vaccination, and vitamin A supplementation.

The preponderance of males with blindness compared with females in this study is in keeping with many previous Nigerian studies.^5^,^6^ Because most of the blind are from the Northern region of the country, gender distribution could also have been influenced by cultural and religious factors. It is unlikely that a female from the North would migrate to the South for socioeconomic reasons.

The majority of the blind in this study realized the importance of education and that it could give them a better future. This is suggested by the large number of respondents who reported that the blind should be educated and also had knowledge of institutions for education of the blind. It is however unfortunate that a majority had never been to these institutions or had any form of vocational training [Table 3]. It was therefore not surprising that street begging was the major occupation reported by the blind in this study. This phenomenon suggests a failure in the social rehabilitation of the blind. When blind people do not have access to formal education or rehabilitation centres or are not rehabilitated properly, they are not equipped for any work. Therefore, the milieu of stigma and discrimination lead to begging, which is the easiest thing to do under the circumstances. Although many beggars earn more than the National minimum wage per day of 250 naira (Federal Government of Nigeria, National Minimum Wage Amendment Act 2000) or equivalents of about 1.5 American dollars, they still reported a desire for a change of job, suggesting that begging was still objectionable to them [Table 3].

Studies of marriage and family life among the blind report widely varying outcomes. Yet, they do suggest that they have difficulties getting married and running their families.^24^ Marital status in this study indicates that most (80%) of the cohort was married, with the majority having been married for longer than 10 years. This result may suggest that the blind had no difficulties getting married; most actually found a partner by themselves and more than half were in polygamous relationships that produced children. From our outcomes, it can be concluded that the blind enjoyed a reasonable degree of family life and relationships.

Reports on independent daily living of the blind also vary greatly.^4^,^19^ The majority of the blind in this study lived with their spouses or relatives who took care of their cooking, basic hygiene, financial matters and mobility. Although a majority of the blind reported not receiving enough support from family members, they may actually be referring to financial support because family members took care of their domestic needs.

Family members involved in the care of the blind should be encouraged, with training and counselling directed at this group, to aid the rehabilitation process and mitigate psychosocial dysfunction.

The prevalence of probable psychological morbidity in this study of 51% was high compared with 28% and 41% found in two studies using SRQ in mothers of sickle cell disease children and mothers experiencing family violence, respectively.^25^,^26^ Although these studies were carried out in the same environment with similar methodology, the differences in the precipitating event (e.g., abuse, child with pathology versus blindness) may explain the difference. There is general consensus on the fact that blindness has a deleterious psychosocial effect on the afflicted people. It was not surprising that many in our cohort scored high on the SRQ.

The large number who reported feeling happy despite blindness may be related to religious–cultural factors, which tend to inhibit vocal or unrestricted expression of emotional distress.

Approximately half of them entertained fears about the possibility of their children going blind [Table 2]. Those with a family history of blindness (16%) need special attention to allay this fear. The risk of a genetic cause of blindness or a recurrent familial risk factor and beliefs or traditional practices should also be entertained.

The hope of a cure in the presence of longstanding irreversible blindness also concurs with the strong religious background in this society. However, this may also indicate ignorance, which unscrupulous individuals may exploit, resulting in greater financial losses and/or psychological distress for the blind.

Comparisons of the two groups in terms of demographic, socioeconomic and family structure and functioning were performed [Table 4]. The BA group was gainfully employed, none was involved in begging and they received orthodox eye treatment. This differed from the SBC group probably due to the western education and high rate of vocational training in the BA group, which affirms the important role of education in the social adjustment of the blind. The BA group reported greater family support, a greater likelihood of being happy and were less likely to be identified as probable cases of psychological disorder based on SRQ score ≥5. This difference may also be traced to the higher socioeconomic status (because of education and employment) of the BA subgroup compared with the SBC group.

However, fewer subjects in the BA group were married compared with the SBC group, likely implying that the BA group probably found it more difficult finding a partner compared with the SBC group. The role of culture and tradition may also be critical as it is possible that marriage contracts in Southwest Nigeria, where the entire BA subjects come from, may be more stringent than those of the Northern region, where the SBC subjects come from.

A drawback of this study is the small sample size of the cohort, which may not be indicative of the entire population of the blind in the country. In an effort to enlist a reasonable sample size, we used the leaders of each group to assist in the enrolment of subjects. The leaders would likely be more successful in encouraging members of their group to participate than a stranger (study personnel). Additionally, the subjectivity of the verbal questionnaires and the desire of some subjects to “please” the study personnel may confound the outcomes of verbal questionnaires. The conversion of the questionnaire to a regional language may also lack some of the nuances of the original questionnaire that may reduce effectiveness. To somewhat mitigate these drawbacks, we used a validated, standardized questionnaire for portions of this study (SRQ). Furthermore, we used study personnel fluent in the local language to conduct the questionnaire. Lastly, we believe that the lack of visual cues (the study personnel’s reaction) during questioning may actually lead to greater impartiality of the respondents.

In this study, we found that the majority of the blind people were married with children, had longstanding blindness, no formal education or vocational training and therefore lived by street begging. They appeared to have a reasonable degree of social and family interaction and support; yet, there was a high rate of probable psychological dysfunction.

Onset of blindness for the majority was probably in childhood, indicated by corneal disease being the most frequent cause of blindness and the large gap between mean duration of blindness and mean age of the cohort. This implicates inadequate community access to eye care services, which causes a high prevalence of blindness in those at the fringes of the socioeconomic sphere with little or no education and no job, which makes them very vulnerable.

Our study underscores that blindness in a majority of the cases in this population of people was probably preventable. Formal rehabilitation was never undertaken by the majority. Most of the individuals considered begging as a means of survival but wished for other occupations. The blind require assistance in social rehabilitation, preventive ophthalmology, community health care and psychosocial care.
